# Clinical Biofluid Assays for Prostate Cancer

**DOI:** 10.3390/cancers16010165

**Published:** 2023-12-28

**Authors:** Talaibek Borbiev, Indu Kohaar, Gyorgy Petrovics

**Affiliations:** 1 Center for Prostate Disease Research, Murtha Cancer Center Research Program, Department of Surgery, Uniformed Services University of the Health Sciences, Bethesda, MD 20817, USA; tborbiev@cpdr.org (T.B.); indu.kohaar@nih.gov (I.K.); 2 Henry M. Jackson Foundation for the Advancement of Military Medicine, Inc., Bethesda, MD 20817, USA; 3 Division of Cancer Prevention, National Cancer Institute, National Institutes of Health, Rockville, MD 20850, USA

**Keywords:** prostate cancer, liquid biopsy, blood-based and urine-based biomarkers

## Abstract

**Simple Summary:**

Prostate cancer (PCa) is the most frequent malignancy, and the second leading cause of cancer-related death, in men in the United States. PCa detection has been largely determined by the widely used prostate-specific antigen (PSA) blood test, followed by biopsy for a definitive diagnosis. Over the past years, more refined and more specific blood-based assays, as well as urine-based PCa tests, have driven remarkable progress in PCa detection and prognosis. Urine-based “liquid biopsies” provide minimally invasive, or non-invasive, options for the early detection and risk stratification of PCa. This mini review highlights current advancements and future perspectives.

**Abstract:**

This mini review summarizes the currently available clinical biofluid assays for PCa. The second most prevalent cancer worldwide is PCa. PCa is a heterogeneous disease, with a large percentage of prostate tumors being indolent, and with a relatively slow metastatic potential. However, due to the high case numbers, the absolute number of PCa-related deaths is still high. In fact, it causes the second highest number of cancer deaths in American men. As a first step for the diagnosis of PCa, the PSA test has been widely used. However, it has low specificity, which results in a high number of false positives leading to overdiagnosis and overtreatment. Newer derivatives of the original PSA test, including the Food and Drug Administration (FDA)-approved 4K (four kallikreins) and the PHI (Prostate Health Index) blood tests, have higher specificities. Tissue-based PCa tests are problematic as biopsies are invasive and have limited accuracy due to prostate tumor heterogeneity. Liquid biopsies offer a minimally or non-invasive choice for the patients, while providing a more representative reflection of the spatial heterogeneity in the prostate. In addition to the abovementioned blood-based tests, urine is a promising source of PCa biomarkers, offering a supplementary avenue for early detection and improved tumor classification. Four urine-based PCa tests are either FDA- or CLIA-approved: PCA3 (PROGENSA), ExoDX Prostate Intelliscore, MiPS, and SelectMDx. We will discuss these urine-based, as well as the blood-based, clinical PCa tests in more detail. We also briefly discuss a few promising biofluid marker candidates (DNA methylation, micro-RNAs) which are not in clinical application. As no single assay is perfect, we envision that a combination of biomarkers, together with imaging, will become the preferred practice.

## 1. Introduction

PCa is the second most prevalent cancer, with nearly 1.4 million new cases and 375,000 deaths globally, making it the fifth leading cause of cancer death among men in 2020 [1,2]. Specifically in the United States, PCa incidence for 2023 was predicted to be 288,300 new cases and 34,700 deaths [3]. When evaluated across 36 nations, countries in Africa reported the highest mortality associated with PCa [4]. It was noted that the incidence of this disease is markedly higher in African and African American populations, often manifesting in a more aggressive and lethal form. One hypothesis attributing to these racial disparities is the presence of genomic variations. Germline susceptibilities specific to various races have been shown through genome-wide association studies, suggesting differential genomic architecture amongst diverse racial groups [5,6,7]. For instance, African men display a genetic risk score approximately 2.18 times higher than European men, whereas East Asian men exhibit a risk 0.73 times lower than European men [8].

Remarkably, PCa differs from many other malignancies because a large fraction of prostate tumors is indolent with a relatively low metastatic potential. Consequently, many patients may remain asymptomatic and free from complications for prolonged periods of time. Even in cases with metastatic spread, effective management can extend the survival rate. However, if the cancer cannot be controlled, it will progress rapidly, leading to multiple adverse symptoms and death. Therefore, monitoring the status of developing PCa is essential for its treatment.

Early detection strategies for PCa have been prominently dependent on the PSA blood test for decades. Another commonly used screening method for PCa detection is digital rectal examination (DRE), usually performed after an elevated serum PSA level is detected. However, neither the PSA test nor DRE provide enough information to diagnose PCa or to assess its risk of becoming an aggressive cancer. Abnormal results of these tests, however, warrant diagnostic prostate biopsies for pathological examination and, if positive, cancer staging. Cancer-positive diagnostic biopsies are still the only accepted way to diagnose PCa.

A brief introduction of the more recent biofluid-based clinical tests will be reviewed here (Table 1 and Table 2). Two additional blood tests were introduced to clinical use that are better than PSA alone. The PHI test measures three PSA forms in patients with an initial serum PSA of 4–10 ng/mL (“gray zone”). It performs well in predicting the presence of PCa (AUC of 0.7) and similarly well for the presence of high-grade PCA (GS ≥ 7). The four-kallikrein (4K) score, measuring four kallikreins, combined with clinical information, is used to estimate the likelihood of high-grade PCa on biopsy (AUC of about 0.8).

Urine-based PCa tests approved for clinical use include the PCA3 (PROGENSA) test, which measures mRNA levels of *PCA3*, a non-coding PCa-specific gene, in post-digital rectal exam (post-DRE) urine specimens. For the prediction of first or repeat biopsy results, an AUC of about 0.75 was reached.

The Michigan Prostate Score (MiPS) test combines serum PSA with the mRNA expression levels of the abovementioned *PCA3* gene, and the *TMPRSS2-ERG* gene fusion (specific for PCa), in post-DRE urine specimens. It predicted the presence of PCa in biopsies with an AUC of about 0.75.

A more recent urine-based test, the ExoDX Prostate Test (EPI score), uses mRNA from exosomes present in regular (not post-DRE) urine specimens and measures the level of the *PCA3* and *ERG* genes. It predicts cancer-positive and high-grade cancer-positive biopsies with an AUC of 0.71–0.76 range.

Finally, the SelectMDx test measures mRNA levels of the *HOXC6* and *DLX1* genes in post-DRE urine specimens. It achieved an AUC of 0.79–0.86 for predicting high-grade PCA on biopsy specimens.

## 2. PSA-Related Blood-Based Assays

### 2.1. Serum PSA

PSA, a protein found in blood, is produced by both normal and malignant prostate epithelial cells within the prostate gland. PSA is a 34-kDa glycoprotein comprising 240 amino acids, exclusively produced by prostatic epithelial cells. As a serine protease from the kallikrein gene family, it shares similarities with human glandular kallikrein and exhibits activities like chymotrypsin, trypsin, and esterase. Predominantly in serum, PSA binds with alpha 1-antichymotrypsin, and its production seems to be regulated by circulating androgens acting through the androgen receptors. The role of serum PSA as PCa biomarker was first discovered in 1979 [9,10,11].

The PSA test determines the amount of PSA in nanograms per milliliter (ng/mL) of blood. Historically, a PSA reading below 4 ng/mL is considered normal, while higher levels warrant an additional PSA test and DRE. The Food and Drug Administration (FDA) first endorsed the PSA test in 1986 to track prostate cancer in already diagnosed patients. In 1994, the FDA approved the PSA test to be used in conjunction with DRE to aid in the detection of prostate cancer in men. Periodic PSA screening for men between 55 and 69 years old is suggested but should be individualized, with consideration for potential benefits and harms, due to the low specificity of the serum PSA test. Aside from PCa, benign conditions like prostatitis (inflammation of the prostate) and benign prostatic hyperplasia (BPH) (an enlarged prostate) can also elevate PSA levels. In addition, PSA levels can also change due to age, prostate size, trauma, lifestyle, and certain medications. Even though the PSA test has its limitations, its utility in early disease detection and post-treatment monitoring remains valuable.

### 2.2. Prostate Health Index (PHI)

While the screening of total PSA remains contentious due to its limited specificity in detecting clinically significant PCa, advances in biomarker research have attempted to refine this diagnostic approach. The low specificity of the PSA test may result in unnecessary biopsies leading to the detection of no tumors, or indolent tumors that would not have caused harm during the patient’s lifetime. Given the limitations of the PSA test, a composite metric called the Prostate Health Index (PHI) was proposed. PHI combines the total and free PSA with [−2] proPSA (p2PSA), using the formula p2PSA/(free PSA) × √PSA as a “PHI score”. PSA in the blood is usually bound to other proteins but tends to circulate as “free” PSA in the case of benign prostatic hyperplasia (BPH) or PCa. A ratio of free PSA to total PSA level is used for prostate disease assessment. PSA precursors were found to be a more specific serum marker for PCa. In a study involving 1091 serum samples from patients subjected to prostate biopsies, these precursors emerged as potent predictors of PCa, especially in the PSA range of 2 to 4 ng/mL [12].

One precursor, the [−2] proenzyme PSA, displayed an ability as a predictor of PCa in a cohort of 123 men scheduled to undergo prostate biopsies [13]. The use of this precursor and the free PSA to total PSA ratio enhances the interpretative power of elevated PSA levels, increasing the likelihood of identifying PCa through biopsies. Specifically, the PHI score is suggested for men aged 50 or above, with total PSA results between 4 and 10 ng/mL and negative DRE findings.

PHI was approved by the FDA and is now commercially available in regions including the USA, Europe, and Australia. Notably, in a multicenter prospective trial involving 658 men, PHI displayed superior diagnostic capabilities compared to its individual components, with an AUC of 0.708 [14]. Moreover, another study with 506 participants underscored PHI’s ability to considerably reduce biopsy procedures (36.4% vs. 60.3%) in the control group [15]. Additionally, in a study of >700 patients, while comparing PHI score and other metrics like BPH-associated free PSA, tPSA, fPSA, and p2PSA, PHI exhibited significant increases in PCa predictive value and specificity of PHI [16].

A comprehensive meta-analysis of sixty studies, including 14,255 individuals, illustrated PHI’s better PCa diagnostic efficacy over PSA. Pooled results unveiled that PHI had sensitivities of 0.791 and aspecificity of 0.625 for PCa detection, with clinically significant PCa detection sensitivity being 0.625 and specificity being 0.569 [17]. In the meta-analysis of diagnostic accuracy of PSA for the detection of PCa in 14,489 symptomatic patients and a control group of 9459 men (normal DRE and PSA <4 ng/mL for 7 years), pooled data found the sensitivity of PSA to be 0.93, with a specificity of 0.20, for symptomatic patients, confirming its known high sensitivity but very poor specificity [18,19].

PHI performs as a promising diagnostic biomarker with enhanced specificity and accuracy in detecting PCa, especially its clinically significant forms. By incorporating PHI into clinical practice, current diagnostic gaps could be diminished, offering patients more accurate screening and subsequently better treatment choices.

### 2.3. 4Kscore

The 4Kscore (developed by OPKP Health, Inc.) is a follow-up blood test performed if the patient has abnormal PSA and DRE results, to detect indolent disease and prevent unnecessary biopsies. This test analyzes four prostate-specific biomarkers (total PSA, free PSA, intact PSA, and human kallikrein 2 [hK2]), along with the patient’s clinical information (age, biopsy history, DRE result) via a complex algorithm to produce a final score. Several studies confirmed the usefulness of the abovementioned biomarkers for PCa prediction. In a study involving 161 patients scheduled for radical prostatectomy, hK2 levels correlated positively with the pathologic stage of clinically localized PCa [20]. Similarly, serum specimens from 577 patients, treated with radical prostatectomy, showed that increased concentrations of hK2 in the blood were significantly associated with unfavorable features of PCa [21]. A different study emphasized hK2’s superiority over PSA in accurately identifying poorly differentiated (G3) tumors, which is paramount for clinical decision-making [22]. Sera from 122 patients with PCa, graded from G1 to G3, was studied for total PSA, free PSA, and hK2 readings. hK2 significantly improved the identification of poorly differentiated (G3) tumors compared with PSA, ultimately allowing for better clinical decision-making [22].

The 4Kscore test is approved by the FDA and included in current USA, Canadian, and European PCa early-detection guidelines. The accuracy of the test was demonstrated in several validation studies. In a study of 1012 men scheduled for prostate biopsy, the 4Kscore showed better diagnostic performance in detecting significant PCa than standard of care, with an AUC of 0.82, and the authors stated that up to 36% of biopsies could be avoided or delayed [23]. In a prospective trial of 366 enrolled men (56% African American, 36% Grade Group 2 or higher), the 4Kscore test showed better discrimination (AUC 0.81 versus 0.74) and clinical usefulness than the base model [24]. In a nested case-control study of 12,542 men, followed for >15 years, PSA and 4Kscore were measured to increase the specificity of screening for lethal PCa at an early stage. The 4Kscore significantly enhanced the prediction of metastasis compared with PSA alone (PSA > 2 ng/mL). [25]. A study involving 11,506 men followed for 20 years was undertaken to determine the long-term risk of PCa mortality using the 4Kscore. In men with elevated PSA (≥2.0 ng/mL), predictive accuracy was improved by the 4Kscore compared with PSA alone (0.80 versus 0.73). Also, it was determined that men with an elevated PSA but a low 4Kscore can be put under surveillance rather than undergoing invasive biopsy [26].

The 4Kscore test proves to be a useful tool in deciding if a patient needs to go through a biopsy procedure after repeated abnormal PSA readings and abnormal DRE findings (Table 1).

## 3. Urine-Based Biomarkers

Urine is a promising liquid biopsy choice, especially for PCa, because prostate manipulation enriches it with PCa cells, exosomes, DNAs, RNAs, proteins, and small molecules, which could be used as biomarkers. In comparison with biopsy cores, urine sampling provides a more representative picture of the significant spatial heterogeneity within the patient’s prostate. Several urine-based tests are available: the PROGENSA PCA3 assay, the MiProstate Score assay, the ExoDx™ Prostate IntelliScore test, and the SelectMDx test (Table 1). Even though post-DRE or DRE urine specimens are boosted with prostate secretions, and therefore preferable for following prostate biomarker analysis, non-invasive, non-DRE urine-based gene panels are emerging, with the ExoDx™ Prostate Test being CLIA-approved [27,28,29,30] (Table 2).

### 3.1. PCA3 (PROGENSA)

The PROGENSA *PCA3* Assay was the first FDA-approved urine-based PCa molecular test in 2012 [31]. It informs the decision on repeat biopsy for men with a previous negative biopsy and with an elevated PSA. Its primary function is to detect the overexpression of the *PCA3* gene over PSA to generate a *PCA3* score in post-DRE first-catch urine specimens.

This assay is particularly recommended for men aged 50 and older, who have had one or more previous negative prostate biopsies. The *PCA3* score test is dependent on the cutoff value, and caution should be used while determining “positive” vs. “negative” test results [32].

If the reported *PCA3* score is below the cutoff of 25, the result is considered negative; if more than 25, it is positive. A negative result is correlated with a decreased likelihood of a positive biopsy. Due to normal assay variability, *PCA3* scores between 18 to 31 could yield different results, so caution should be exercised while interpreting test outcomes in this range. The *PCA3* score should be used in conjunction with other patient data to decide if the patient needs a repeated biopsy.

*PCA3* is a prostate-specific gene that is highly overexpressed in prostatic tumors in comparison to non-neoplastic prostatic tissue of the same patients [33], permitting its detection in cancer cells shed into urine after DRE. *PCA3* expression, which is 60- to 100-fold greater in cancerous than in benign prostate tissues, gives the gene a cancer specificity, which is not the case for PSA [34]. In addition, factors that are known to change PSA levels (BPH, prostate volume, age, inflammation, trauma) do not appear to affect *PCA3* levels.

In a prospective validation study of 859 patients, with a mean age of 62 years, a specificity of 76% and a sensitivity of 50%, with an AUC of 0.68, is shown for PCA3 (cutoff at 35 copies/copy of PSA mRNA). The data obtained demonstrated that *PCA3* improved PPV (80%) for the initial biopsy group and NPV (88%) in the repeat biopsy group, thus reducing the burden of prostate biopsies among men undergoing a repeat prostate biopsy [35].

For distinguishing between cancer and non-cancer, a *PCA3* cutoff at 35 copies/copy of PSA mRNA showed a specificity of 76% and a sensitivity of 50%, with an AUC of 0.68. Almost no overlap was detected when comparing cancer versus BPH, confirming the specificity of the *PCA3* score test. The *PCA3* score test is particularly important when a negative biopsy appears following an elevated PSA. In such men, *PCA3* score test prediction for PCa is significantly higher than serum PSA testing [36].

To investigate the association between urine *PCA3* levels and prostate tumor pathology from whole-mount radical prostatectomy (RP) specimens, post-DRE urine samples of 72 patients diagnosed with PCa were collected. A clear link between the *PCA3* score and total tumor volume was detected. Moreover, patients with extraprostatic tumor extension showed higher median *PCA3* scores than patients without this complication (48.8 versus 18.7). For this, a *PCA3* score cutoff of 47 demonstrated 94% specificity, 80% positive predictive value, AUC of 0.90, when combined with serum PSA and biopsy Gleason score [37]. *PCA3* gene expression levels were shown to be an important tool in augmenting serum PSA testing, particularly in cases when raised PSA levels were due to prostatitis rather than to cancer on initial biopsy, in men under surveillance with Gleason score 6 and less, and in men with prostate cancer and normal PSA levels [38].

### 3.2. MiProstate Score (MiPS)

The MiPS assay is a multiplex evaluation of *T2-ERG* gene fusion, *PCA3*, and serum PSA (KLK3). It is commercially available through the University of Michigan MLabs. The MiPS assay tests for the presence of two prostate cancer biomarkers in post-DRE urine specimens: *PCA3* gene mRNAs, found to be overactive in 95% of all PCa-s, and *TMPRSS2: ERG* mRNAs, which is also a strong indicator of PCa.

The MyProstateScore, produced using MiPS assay, has been verified to ameliorate the detection of clinically significant (Gleason grade [GG] ≥ 2) PCa, when compared to risk calculators based on PSA levels.

In a validation study of MiPS feasibility on 1525 patients, the MiPS set at a threshold of 10 demonstrated 97% sensitivity and 98% negative predictive value for GG of 2 or higher prostate cancer. Using MiPS testing could have eliminated 387 unnecessary biopsies (33%), thus presenting a less invasive and more cost-efficient alternative to accustomed procedures after screening with PSA [39]. The combination of T*2: ERG* and *PCA3* urinary levels can minimize unnecessary biopsies, while maintaining high sensitivity for detecting aggressive PCa, and eventually lead to potential savings in healthcare costs [40].

Observed *ERG* overexpression was linked to *TMPRSS2: ERG* fusion, which was discovered through a bioinformatics approach. Cell line experiments have proven that the androgen-responsive promoter elements of *TMPRSS2* are involved in the overexpression of *ERG* in PCa [41]. In addition, a significant correlation between *ERG* and TMPRSS*2: ERG* expression in PCa patients was found, with these transcripts being measurable in their post-DRE urine specimens [42]. In a bigger PCa patients’ cohort (n = 114), *ERG* overexpression has been detected in malignant prostate epithelial cells [43]. In a meta-analysis study of the inter-racial distribution of *TMPRSS2: ERG* fusions in PCa, the highest occurrence was detected in men of European ancestry (49%), followed by men of Asian (27%) and then African (25%) ancestry. The lower incidents of TMPRSS*2*: *ERG* fusions in men of African descent suggests the implication of distinct genomic mechanisms [44]. Interestingly, the presence of *TMPRSS2-ERG* is inversely related to aggressive PCa in men of African descent, showing an association with lower-grade diseases [44,45].

In a study involving 291 patients at risk of PCa or under active surveillance, the link between TMPRSS*2: ERG*, *PCA3*, PSA density, genetic variants, and androgenic status and biopsy pathological findings, was examined. Notable augmentation in detecting high-risk localized PCa was achieved, with an AUC of 0.74 [46].

### 3.3. ExoDx™ Prostate IntelliScore

The ExoDx™ Prostate Test is a urine-based liquid biopsy test, indicated for men with serum PSA of 2–10 ng/mL, who are 50 years of age and older and may be considering a biopsy. One of the unique attributes of this assay is that it uses a regular urine sample, not a post-DRE sample (it requires no DRE procedure), so it is completely non-invasive. Another unique aspect of this test is that it uses RNA obtained from exosomes, small lipid vesicles which are generated by all cells, and which protect the content of the vesicle from the environment. The RNA isolated from the urinary exosomes is intact, and no negative effect of the harsh environment of urine is observed. The test is CLIA-approved, and is covered by major insurance, including Blue Cross, Blue Shield, and Medicare.

The assay examines the expression levels of the *ERG*, *PCA3*, and, as a control, the *SPDEF* gene. The ExoDx Prostate Test offers a risk score (EPI score) to determine a patient’s chances for clinically significant PCa (with a Gleason score of ≥7 on biopsy). Recognized by the National Comprehensive Cancer Network (NCCN) guidelines, the test’s clinical efficacy is validated at a cut-point of 15.6.

In a prospective, blinded, randomized, multisite clinical study, urine samples were collected from 1049 men (≥50 years old) with a PSA of 2–10 ng/mL being considered for a prostate biopsy. Clinical outcomes, time to biopsy, and pathology were assessed among low (<15.6) or high (≥15.6) EPI scores. The results revealed that those with low EPI scores postponed their first biopsy and remained at a low pathologic risk status for 2.5 years following the study [47].

In a meta-analysis of three independent studies involving 1212 men, with a median age of 63 years old, median PSA of 5.2 ng/mL, who were going through their initial biopsy, the EPI AUC (0.70) outperformed PSA (0.56) and the Prostate Cancer Prevention Trial Risk Calculator (PCPT-RC) (0.62) in differentiating between GG2, GG1, and benign histology. A cutoff set at 15.6 would circumvent 30% of unnecessary biopsies, with an NPV of 90% [48].

In another study of 2066 men over 50 years old scheduled for an initial biopsy, with a PSA between 2 and 10 ng/mL, the EPI score was found to outperform the multivariate risk calculators. Of these men, 310 proceeded to radical prostatectomy, of whom 111 patients had GG1 at biopsy and would have been potential candidates for active surveillance [49].

A study of 1064 patients validated the potency of the ExoDx Prostate IntelliScore combined with the standard of care (PSA level, age, race, and family history). The AUC of this combined approach was 0.77 versus a standard of care AUC of 0.66 [50].

Finally, in a study of 503 patients, it was further demonstrated that the EPI score can predict ≥GG2 PCa at initial biopsy and can postpone unnecessary biopsies at a higher precision than existing risk calculators and standard clinical data [51].

### 3.4. SelectMDx (MDx Health)

The SelectMDx test is an advanced diagnostic procedure that quantifies the presence of two specific genes linked to aggressive forms of PCa. The measurement of these PCa-specific genes in combination with traditional clinical risk factors—such as age, serum PSA level, prostate volume, family history of PCa, and the outcome of a DRE result—compose a patient’s SelectMDx personalized risk score.

The SelectMDx risk score provides insight into whether PCa is likely to be found upon initial biopsy. Therefore, an individual with a clearer understanding of their SelectMDx risk score is in a better position to decide on the potential benefits of undergoing an early biopsy. The SelectMDx test is included in the National Comprehensive Cancer Network (NCCN) Guidelines for Prostate Cancer Early Detection.

*HOXC6* expression, measured through mRNA and protein levels, was found to be prostate-specific and correlated with high serum PSA levels, Gleason score, and TNM stage, implicating this biomarker for PCa detection and metastatic prognosis. Furthermore, it was observed that a reduced expression of *HOXC6* was associated with limited proliferative, migratory, and invasive capabilities of PCa cells [52].

*HOXC6* expression amount can be detected in urine. Urinary *HOXC6* mRNA levels complemented with *DLX1* mRNA expression were used in a validation study of 1955 patients prior to an initial prostate biopsy. The most practical clinical model included urinary *HOXC6* and *DLX1* mRNA levels, patient age, DRE results, and PSA density to differentiate higher-grade PCa in patients with a PSA less than 10 ng/mL. This model achieved an AUC of 0.82, 89% sensitivity, 53% specificity, and a negative prediction value of 95%. Thus, this two-gene urine panel demonstrated its feasibility to detect clinically significant PCa prior to initial biopsy [53].

In two prospective studies, post-DRE urine samples were collected from 907 patients for *HOXC6* and *DLX1* mRNA profiling prior to initial biopsy. *HOXC6* and *DLX1* mRNA levels were potent predictors of high-grade PCa, with an AUC of 0.90 in the validation cohort and 0.86 in the training cohort. This tri-gene panel, together with traditional clinical risk factors, offered better risk stratification than current clinical methods [54]. Since *HOXC6* upregulation was found in all primary, metastasized, and castration-resistant PCa, it could be used in early detection as well as disease progression [55].

## 4. Additional Biofluid Markers in Development

### 4.1. DNA Methylation

The DNA methylation status of certain genes may differ significantly between tumors and normal tissue in PCa (Table 3). In a study of 24 selected markers in 213 prostate biopsy cores from 104 patients, this marker panel determined cancer cores and benign cores from non-cancer patients with 100% sensitivity and 97% specificity [56].

In a study of biopsy and urine specimens from 161 patients, promoter methylation of two gene panels was evaluated using MethyLight methodology. In tissue samples, methylation panels detected PCa with an AUC of 0.977, whereas in urine samples, an AUC of 0.98 was detected. Thus, quantitative gene panel promoter methylation in either tissue biopsy or in urine might be a clinically useful tool for PCa detection and risk stratification for disease aggressiveness [57].

The potential of *RARβ2* methylation as a urine biomarker was evaluated in a meta-analysis focusing on the methylation frequency of *RARβ2*—a parameter hypothesized to be elevated in PCa patients compared to controls. In this study, 777 cases and 404 controls were involved. The analysis indicated that *RARβ2* could be a promising biomarker for both the prevention and diagnosis of PCa [58].

Post-DRE urine samples were collected from 153 patients on active surveillance with a Gleason score of 6 to predict disease progression. A classifier panel with four methylation biomarkers, *APC*, *CRIP3*, *GSTP1*, and *HOXD8,* was identified. This panel proved effective in predicting disease progression in the monitored patients [59].

Methylation biomarkers of a Prostate Cancer Urinary Epigenetic (ProCUrE) study, based on the methylation of two genes (*HOXD3* and *GSTP1*), were analyzed in post-DRE urinary sediments. The ProCUrE assay efficacy was tested in a study involving 408 patients in risk categories ranging from benign to low, intermediate, and high-risk PCa. The ProCUrE assay displayed a marked improvement in diagnosing PCa and identifying patients with clinically significant disease [60].

### 4.2. Micro-RNAs (miRNAs)

miRNAs are short non-coding RNAs (18–25 nucleotides) that are involved in post-transcriptional regulation of target genes. miRNAs were detected in urine as a free form, in cell sediments, or in exosomes. The utility of urine-circulating miRNAs as a potential biomarker of PCa was evaluated (Table 3).

Initial screening of 754 miRNAs from prostate tissues of both cancerous patients (n = 56) and non-cancerous patients (n = 16) using the TaqMan low-density array identified over 100 miRNAs with deregulated expression in PCa. Specifically, the abundance of miR-148a and miR-375 in urine was identified as a biomarker of PCa. Combined analysis of miR-148a and miR-375 was sensitive and specific (AUC = 0.79 and 0.84) [61].

Five miRNAs that were consistently deregulated in PCa (miR-21, miR-141, miR-214, miR-375, and let-7c) were analyzed in urinary pellets from 60 PCa patients and 10 healthy subjects by qRT-PCR. Significant upregulation of three miRNAs (miR-21, miR-141, and miR-375) and downregulation of one miRNA (miR-214) were observed. A combined miR-21 and miR-375 panel presented a strong potential to differentiate between the two groups, with an AUC of 0.872 [62].

A model incorporating five urine miRNAs (miR-151a-5p, miR-204-5p, miR-222-3p, miR-23b-3p, and miR-331-3p) and serum PSA, termed pCaP, was developed after examining 45 selected miRNAs in 215 PCa patients. This model significantly predicted the time to biochemical recurrence, indicating its potential in refining risk stratification and individualizing treatment plans [63].

Two novel three-miRNA diagnostic and prognostic models (miR-222-3p/miR-24-3p/miR-30c-5p and miR-125b-5plet-7a-5p/miR-151-5p) were examined using urine samples from 29 BPH and 215 PCa patients. The diagnostic model (miR-222-3pmiR-24-3p/miR-30c-5p) effectively differentiated BPH from PCa with an AUC of 0.95, while the prognostic model (miR-125b-5plet-7a-5p/miR-151-5p) projected time to biochemical recurrence after radical prostatectomy independently of other clinical parameters [64].

Validation of the previously described model, miR-222-3p/miR-24-3p/miR-30c-5p, was performed on urine samples from 758 patients with clinically localized PCa, 289 non-cancer controls with BPH, and 233 patients undergoing initial TRUS-guided prostate biopsy. This model distinctively identified PCa in comparison to BPH, with an AUC of 0.84. Moreover, it surpassed PSA in forecasting TRUS biopsy outcomes in patients within the diagnostic gray zone [65].

### 4.3. Other RNA-Based Biomarkers (PSGR, PCGEM1, PSMA) in Development

*PSGR*, a gene with homology to the G protein-coupled odorant receptor family, has been identified as a PCa-specific gene, which is overexpressed in PCa but shows very little or no expression in prostatic epithelial cells of matched normal specimens. Overexpression of *PSGR* in PCa provides a foundation for the potential of *PSGR* as a PCa biomarker [70,71]. It was found that, compared to normal and BPH tissues, the expression of *PSGR* increased significantly in PIN and prostate tumors (approximately 10-fold), especially in early prostate cancer, suggesting *PSGR* has the potential for early PCa detection (AUC of 0.902) [66]. *PSGR* expression was readily detected by qPCR in post-DRE urine sediments of 215 patients for PCa detection [67]. A total of 220 RNA specimens from benign and malignant prostatic epithelial cells of 110 PCa patients were investigated for association of the *PSGR* overexpression in the different PCa pathologic stages. *PSGR* overexpression showed a positive correlation with pT3 and a higher level of serum PSA. Additionally, PCa cells of African American patients demonstrated about a two-fold increase of *PSGR* expression in comparison to Caucasian American patients [72].

A prostate-specific gene, *PCGEM1*, was identified as long non-coding RNA, preferentially expressed in prostate tumors [73], and *PCGEM1* expression was significantly higher in the PCa of African American men than in Caucasian American men [74].

*PCGEM1* expression was found to be markedly downregulated following castration and upregulated upon androgen receptor activation in vivo. *PCGEM1* expression levels were repressed in response to androgen ablation therapy, indicating that PCGEM*1* is a transcript regulated by androgens [75].

In another study focusing on non-DRE urine specimens, collected from patients undergoing diagnostic biopsy, a two-gene panel, consisting of *PCGEM1* and *PCA3*, was used to differentiate high-grade cancer (Gleason score 7–10) from low-grade cancer (Gleason score 6) in a patient cohort with about 30% of African American men. When combined with standard of care variables, this panel notably enhanced the prediction of high-grade cancer at diagnosis compared to using standard of care variables alone (AUC 0.88 vs. 0.80) [68].

Prostate-specific membrane antigen (PSMA) is a transmembrane protein predominantly found in PCa epithelial cells, exhibiting higher expression levels in cancerous cells than in non-cancerous cells. In a study investigating post-DRE urine samples from 154 patients undergoing prostate biopsies, biomarkers consisting of PSMA, *PSGR*, and *PCA3* were used to distinguish between advanced versus indolent PCa, to then determine which patients should undergo biopsy. It was concluded that the *PSMA*, *PSGR*, and *PCA3* scores were significant indicators of PCa, with an AUC of 0.82 [69].

In a further investigation, the diagnostic relevance of five PCa-associated genes (*PSMA*, *Hepsin*, *PCA3*, *GalNAC-T3*, and *PSA*) was evaluated in 44 patients diagnosed with PCa, and 46 patients with BPH were reviewed along with post-DRE urine samples. After measuring the absolute concentration of each gene’s transcript, the diagnostic capabilities of the combined urinary *PSA* and *PSMA* levels were more effective than any singularly considered marker [76].

## 5. Conclusions

Although tissue biopsy remains the gold standard for PCa diagnosis and assessment, liquid biopsy biomarkers offer a supplementary minimally invasive, or non-invasive, avenue for early detection and improved tumor classification. Urine is a minimally (DRE) or non-invasive (non-DRE) liquid biopsy source well-suited for PCa since it is enriched with cancer cells, exosomes, DNAs, RNAs, proteins, and small molecules coming from the prostate. Also, urine-sampling provides a more representative picture of the significant spatial heterogeneity within the patient’s prostate. Although the utility of the PSA test in early disease detection and post-treatment monitoring remains valuable, additional FDA-approved PSA assays, the PHI, and 4Kscore, are available and have higher specificity. Four urine-based PCa tests are either FDA- or CLIA-approved: PCA3 (PROGENSA), ExoDX Prostate Intelliscore, MiPS, and SelectMDx. There are emerging urine-based diagnostic and prognostic gene expression panels, which could potentially be used as new tools for better PCa management.

## Figures and Tables

**Table 1:** Current FDA- or CLIA-approved biofluid-based biomarkers in prostate cancer.

Biomarker Test	Molecular Markers	Approval
**Serum-based**		
Prostate Serum Antigen	PSA	FDA
Prostate Health Index, PHI, Beckman Coulter Inc., Brea, CA, USA	Total PSA, fPSA, p2PSA	FDA
4Kscore, OPKO Health company, BioReference Laboratories Inc., Township, NJ, USA	Total PSA, fPSA, intact PSA, hK2	CLIA
**Urine-based**		
Progensa PSA3 Assay, Hologic, MA, USA	*PCA3*	FDA
ExoDX Prostate (Intelliscore), Exosome Diagnostics Inc., Waltham, MA, USA	Exosomal RNA (*PCA3*, *ERG*)	CLIA
MiPS, MyProstateScore, MCTP, Arbor, MI, USA	*PCA3*, *TMPRSS2-ERG*	CLIA
SelectMDX, MDx Health, Irvine, CA, USA	*HOXC6*, *DLX1*	CLIA

**Table 2:** Urine-based assays: advantages and limitations.

Biomarker Test	Urine Sample	Advantages	Limitations
Progensa PSA3 Assay, Hologic, Marlborough, MA, USA	Post-DRE	Assay score correlates with the likelihood of repeat biopsy	Sample collection requirements, clinical data and risk factors should be considered before decision, certain conditions, procedures and medications may influence assay score
ExoDX (Prostate Intelliscore), Exosome Diagnostics Inc., Waltham, MA, USA	Non-DRE	Non-invasive test, at-home collection kit, assay score correlates with risk for advanced PCa	All clinical data should be considered before decision
MiPS, MyProstateScore, MCTP, Arbor, MI, USA	Post-DRE	Assay score correlates with a need for biopsy	All clinical data required to generate final score and should be considered before decision
SelectMDX, MDx Health, Irvine, CA, USA	Post-DRE	Assay score predicts advanced PCa in biopsy	Sample collection requirements, all clinical risk factors should be considered before decision

**Table 3:** Examples of promising methylation and RNA-based urinary biomarkers in development.

Molecular Target	Patient Cohort, N	Sensitivity %	Specificity%	AUC	Reference
24 gene methylation panel	104	100	97	0.88–0.99	[56]
miR-34b/c, mir193b methylation	161	97	80	0.98	[57]
RARβ2 methylation, meta study	1181	-	-	-	[58]
*APC*, *CRIP3*, *GSTP1*, *HOXD8* methylation	153	-	-	-	[59]
*HOXD3*, *GSTP1* methylation	408	57	97	0.8	[60]
miR-148a, miR-375	277	83	79	0.79–0.84	[61]
miR-21, miR-375, 5 miRNA panel	70–215	-	-	0.87	[62,63]
miR-222-3p/miR-24-3p/miR-30c-5p	249–1047	-	-	0.71–0.95	[64,65]
*PSGR*	146–215	-	-	0.68–0.90	[66,67]
*PCGEM1*, *PCA3*	271	-	-	0.88	[68]
*PSMA*, *PSGR*, *PCA3*	154	-	-	0.82	[69]

## Data Availability

The data presented in this study are available in this article.
